# Thin-film transistor-driven vertically stacked full-color organic light-emitting diodes for high-resolution active-matrix displays

**DOI:** 10.1038/s41467-020-16551-8

**Published:** 2020-06-01

**Authors:** Sukyung Choi, Chan-mo Kang, Chun-Won Byun, Hyunsu Cho, Byoung-Hwa Kwon, Jun-Han Han, Jong-Heon Yang, Jin-Wook Shin, Chi-Sun Hwang, Nam Sung Cho, Kang Me Lee, Hee-Ok Kim, Eungjun Kim, Seunghyup Yoo, Hyunkoo Lee

**Affiliations:** 1 Reality Display Research Section, Electronics and Telecommunications Research Institute (ETRI), Daejeon, 34129 Republic of Korea; 2 School of Electrical Engineering, Korea Advanced Institute of Science and Technology (KAIST), Daejeon, 34141 Republic of Korea; 3 Department of Electronics Engineering, Sookmyung Women’s University, Seoul, 04310 Republic of Korea

**Keywords:** Electronic devices, Organic LEDs

## Abstract

Thin-film transistor (TFT)-driven full-color organic light-emitting diodes (OLEDs) with vertically stacked structures are developed herein using photolithography processes, which allow for high-resolution displays of over 2,000 pixels per inch. Vertical stacking of OLEDs by the photolithography process is technically challenging, as OLEDs are vulnerable to moisture, oxygen, solutions for photolithography processes, and temperatures over 100 °C. In this study, we develop a low-temperature processed Al_2_O_3_/SiN_x_ bilayered protection layer, which stably protects the OLEDs from photolithography process solutions, as well as from moisture and oxygen. As a result, transparent intermediate electrodes are patterned on top of the OLED elements without degrading the OLED, thereby enabling to fabricate the vertically stacked OLED. The aperture ratio of the full-color-driven OLED pixel is approximately twice as large as conventional sub-pixel structures, due to geometric advantage, despite the TFT integration. To the best of our knowledge, we first demonstrate the TFT-driven vertically stacked full-color OLED.

## Abstract (web summary)

To realize organic light-emitting diodes (OLEDs) with improved resolution for display applications, a method for achieving high yield device fabrication is needed. Here, the authors report vertically-stacked transistor-driven full-color OLEDs with photolithography-processed intermediate electrodes.

## Introduction

Organic light-emitting diodes (OLEDs) have been widely used in the main displays of mobile phones, tablet PCs, and TVs. OLEDs are also applied to augmented reality (AR) and virtual reality (VR) devices, owing to their excellent characteristics, which include high efficiency, fast response time, high contrast ratio, thinness, light weight, and high-color gamut^1–6^. Since standard AR and VR devices are either glass- or head-mounted, their screens are close to the eyes, and thus, high-resolution display panels are required^7–9^. However, the complex color-patterning process in OLEDs limits opportunities for OLED display resolution enhancement^10^.

The conventional pixel structure in full-color displays consists of three, laterally arranged sub-pixels that have red (*R*), green (*G*), and blue (*B*) colors. Direct patterning, or white OLEDs with color-filter technology, can be used for *R*, *G*, and *B* sub-pixel patterning in OLED displays^11–13^. In the case of direct patterning, fine-metal masks (FMMs) and complex alignment processes are needed, which limit opportunities for OLED display resolution enhancement^14,15^. In white OLEDs with color-filter technology, low efficiency and high cost can be issues, due to optical losses from the color-filter, alignment between the color-filter and the white OLED, and the complexity of white OLED structures. Consequently, laterally arranged sub-pixel structures decrease the geometric fill-factor, becoming a barrier to achieving high-resolution in AR and VR devices.

Color-tunable OLEDs, created using vertically stacked *R*, *G*, and *B* OLEDs, have been reported^16–27^. These devices can express various colors by controlling each color element, and can be a pixel in OLED displays without using FMMs for *R*, *G*, and *B* patterning, complex alignment, or color-filter processes. Transparent intermediate electrodes are required for color-tunable OLEDs with vertically stacked *R*, *G*, and *B* elements, however, most reported color-tunable OLEDs use a shadow mask process^16–18,21–27^, which is unsuitable for high-resolution displays, for intermediate electrode patterning. To achieve a high-resolution display through a vertically stacked structure, the transparent intermediate electrode should be patterned using photolithography process, rather than through the shadow mask process. However, the photolithography process uses organic solvents, etchants, and high temperatures, which can damage OLEDs. Materials used in OLEDs are generally susceptible to damage by moisture, oxygen, organic solvents, and high temperatures^28–33^; therefore, developing a low-temperature process for forming intermediate electrodes and via holes without damaging the OLEDs, is an important advance required for making thin-film transistor (TFT)-driven, vertically stacked full-color OLEDs, using photolithography process.

In this study, we develop vertically stacked OLEDs resistant to the temperature and solvent damage caused by photolithography processes. To the best of our knowledge, this is the first report of full-color OLEDs fabricated using intermediate electrodes patterned by photolithography process. To prevent solvent penetration during the photolithographic process, we design Al_2_O_3_ thin-film encapsulation (TFE) and SiN_x_ passivation layers for application to OLEDs. In our structure, the *R*, *G*, and *B* OLED units are separated by TFE and the passivation layer, allowing them to be individually controlled by each driving voltage. Based on these process developments, we successfully develop TFT-driven full-color OLEDs, with vertically stacked *R*, *G*, and *B*. We are also able to make the OLEDs show full-color in a single-pixel, by controlling the driving voltage in each of the *R*, *G*, and *B* units.

## Results

### Intermediate layers facilitating photolithography patterning

**Fig. 1:**
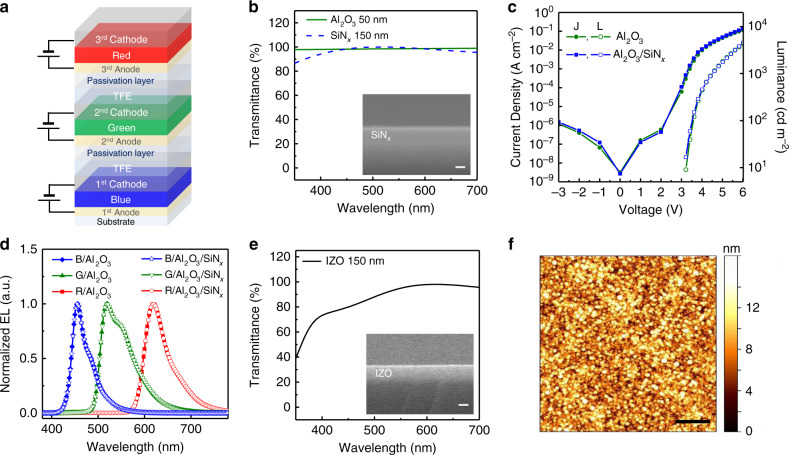
Characterization of intermediate layers for vertically stacked full-color organic light emitting diodes (OLEDs). **a** Schematic of full-color OLED with vertically stacked red (*R*), green (*G*), and blue (*B*). **b** Transmittance spectra for the Al_2_O_3_ (green solid line), and SiN_x_ (blue dashed line) layers, and scanning electron microscope (SEM) image (inset) of the SiN_x_ layer. Scale bar, 100 nm. **c** Current density (*J)–*voltage (*V*)*–*Luminance (*L*) characteristics, **d** Electroluminescence spectra of OLED with Al_2_O_3_ thin film encapsulation, and Al_2_O_3_/SiN_x_ passivation layer. OLED with Al_2_O_3_ (blue line with closed diamond for *B*, green line with closed triangle for *G*, and red line with closed square for *R*), and OLED with Al_2_O_3_/SiN_x_ (blue line with open star for *B*, green line with open down triangle for *G*, and red line with open circle for *R*). **e** Transmittance spectrum and SEM image (inset) of indium zinc oxide (IZO) intermediate electrode. Scale bar, 100 nm. **f** Atomic force microscope image of OLED/Al_2_O_3_/SiN_x_/IZO. Scale bar, 1 μm.

A schematic of the vertically stacked full-color OLED with the *R*, *G*, and *B* primary colors is presented in Fig. 1a, with detailed layer information depicted in Supplementary Fig. 1. The *B*, *G*, and *R* units are the first, second, and third OLED units, respectively. The first anode for the *B* unit was a pre-patterned indium tin oxide (ITO) glass, which has high transparency in the visible region (>85% at 400–700 nm, Supplementary Fig. 2), and was appropriate for bottom emission. Organic layers and cathodes for the OLEDs were created using the thermal evaporation method. The cathodes for the *B* and *G* units were thin, semi-transparent metal, made of aluminum (Al) (2.5 nm)/silver (Ag) (30 nm) to direct green and red light in the bottom direction. The cathode for the R unit was 100-nm-thick Al, which is a reflecting metal. As shown in Supplementary Fig. 2, the Al (2.5 nm)/Ag (30 nm) layer has transparency in the visible region, allowing the letters behind the cathode to be visible, while the 100-nm-thick Al layer showed almost zero transmittance, and the letters are invisible. The Al_2_O_3_ layer was used as a TFE layer, protecting the OLED from moisture and oxygen. The layer has high transparency in the visible region (>98% at 500–700 nm for 50 nm thickness) (Fig. 1b, green solid line), with a refractive index of 1.6 and excellent gas-barrier properties (4.20 × 10^−4^ g m^−2^ per day), as shown in Supplementary Fig. 3.

For the full-color OLEDs with fine patterning adaptation for high-resolution display, photolithography process is most adjustable, allowing the second and third anodes, for the *G* and *R* units, to be patterned on top of the first OLED. However, few studies have reported using photolithography process because OLEDs are easily damaged by the solvents used in the process; for example, Supplementary Fig. 4 shows that the OLED appears to have been damaged by the photoresist (PR) developer, etchant, and PR stripper. Although the Al_2_O_3_ TFE layer protects the OLED, this protection was apparently insufficient to prevent damage from photolithography solvent penetration, probably due to imperfections such as pinholes. This meant that a passivation layer was needed on the TFE layer, to prevent solvent permeation in photolithography processable full-color OLEDs. This passivation layer needs to be deposited at low temperature, as OLED organic layers are vulnerable to temperatures over 100 °C. The passivation layer should also be highly transparent in the visible region, to allow penetration of light from the *R* and *G* units to the bottom direction. SiN_x_ was selected for the passivation layer, as it has high transparency in the visible region (>95% at 500–700 nm, for 150 nm thickness) (Fig. 1b, blue dashed line), and a refractive index of 1.9. It was deposited at low-temperature (100 °C), using plasma-enhanced chemical vapor deposition (PECVD). It was necessary to check the effect of the SiN_x_ passivation layer on the performance of the OLEDs. The current density–voltage–luminance (*J–V–L*) characteristics of the OLEDs were measured, before and after SiN_x_ deposition. As shown in Fig. 1c, the *J–V–L* characteristics of OLED/Al_2_O_3_/SiN_x_ showed almost the same of those for OLED/Al_2_O_3_. The electroluminescence (EL) spectra of *R*, *G*, and *B* OLEDs remained unchanged after SiN_x_ deposition (Fig. 1d). Two color units separated by TFE and a passivation layer can thus be controlled independently.

For the *G* and *R* units, the second and third anodes, which denote the intermediate electrodes, were deposited and patterned. Similar to the passivation layer, the intermediate electrode is processed at low temperature, and should be exhibit a high degree of transparency in the visible region. Indium zinc oxide (IZO) is well known as a transparent electrode that can be deposited at low temperatures, and has been widely used for developing OLEDs. As shown in Fig. 1e, IZO was deposited at 40 °C, in a 150-nm-thick layer. IZO has high transparency in the visible region (>90% at 500–700 nm), with a refractive index of 2.0, and low sheet resistance (~39 Ω sq^−1^).

Electrode and intermediate layer surface roughness characteristics have been presented in Supplementary Fig. 5, which is a 5 × 5 μm^2^ AFM image. Two-dimensional plane views of ITO, IZO, Al_2_O_3_, SiN_x_, Ag, and Al layers were observed. It is important to have smooth surfaces, to reduce leakage current in the OLEDs; each layer was observed to have a smooth surface, with root-mean-square roughness (*R*_q_, nm) values of 2.0, 1.2, 0.4, 1.7, 3.0, and 4.6, for ITO, IZO, Al_2_O_3_, SiN_x_, Ag, and Al, respectively (Supplementary Table 1). An AFM image of OLED/Al_2_O_3_/SiN_x_/IZO, which has an *R*_q_ value of 2.7 nm, is shown in Fig. 1f. Energy level diagram of the intermediate layers (Al_2_O_3_ and SiN_x_) and adjacent electrodes (LiF/Al/Ag and IZO), based on ultraviolet photoelectron spectroscopy (UPS) measurements and reference papers^34–43^ is shown in Supplementary Fig. 6a. The LiF/Al/Ag (4.6 eV), and IZO (4.3 eV) work functions were obtained from UPS spectra in the cutoff region (Supplementary Fig. 6b). As shown in the energy level diagram, Al_2_O_3_ and SiN_x_ have large band gaps, and the locations of their conduction or valence bands were far from the electrode work functions. These results indicate that current leakage is extremely low thereby each OLED can operate independently.

### Photolithography-patterned intermediate electrodes

**Fig. 2:**
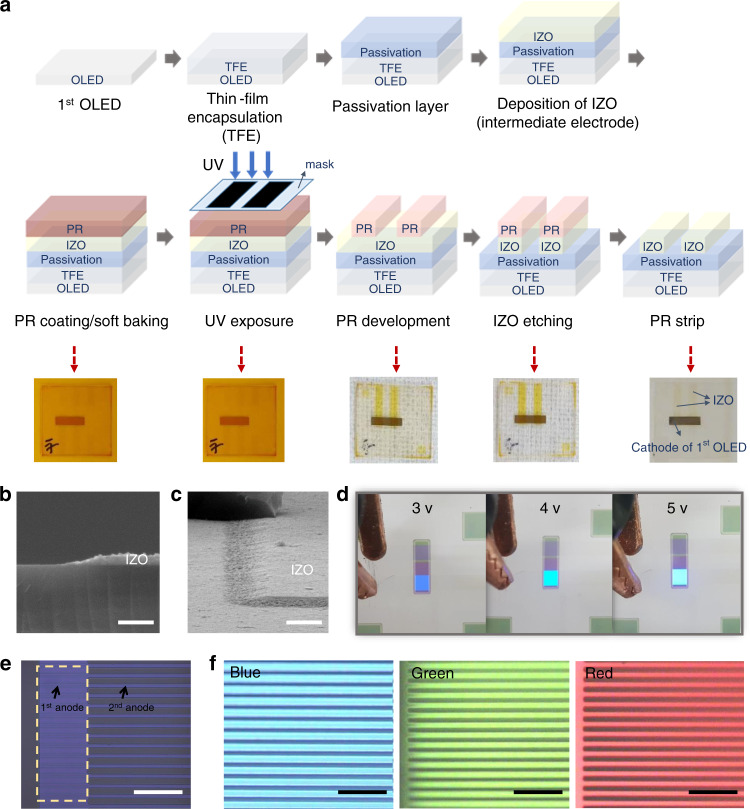
Photolithography-patterned intermediate electrodes. **a** Schematic of the intermediate electrode patterning process and photographs of real device images, for each step. **b** Side view and **c** tilted top view of scanning electron microscope images of the indium zinc oxide (IZO) layer, after patterning. Scale bar, 1 μm. **d** Photographic images of the blue organic light-emitting diode (OLED), with various applied voltages, after second IZO anode patterning by photolithography process. **e** Optical microscope images of fine-patterned second anode with 10 μm linewidths on the first OLED. Scale bar, 100 μm. **f** Blue, green, and red OLED unit luminous images, with 10-μm-patterned electrodes. Scale bar, 100 μm.

A schematic of the intermediate electrode patterning process is shown in Fig. 2a. After the first OLED (*B* unit) was formed, the TFE, passivation layer, and intermediate IZO layer were deposited in succession onto the first OLED, and the photolithography process was continued for patterning of the IZO electrode. The PR mask was made by PR coating, soft baking, UV exposure, PR development, and hard baking. The exposed areas were etched by dipping the OLED into the IZO etchant. Patterned IZO was obtained after removing the PR mask using PR stripper. Actual device images are shown underneath the schematic in Fig. 2a, for each photolithography step. Unlike the previous OLEDs, which had been damaged by the photolithography solutions (Supplementary Fig. 4), the OLED with SiN_x_ passivation layer was observed to be stable, even after all the photolithography processes had been completed.

The patterned IZO electrode was confirmed using scanning electron microscopy (SEM), and the side (Fig. 2b) and tilted top views (Fig. 2c) of the IZO layers, after the patterning processes, can be observed. The side view indicates that the IZO thickness remained unchanged after the patterning process, and etching had only occurred where required. The patterned IZO had a negligible slanted side, compared to that developed by a shadow mask method, where the shadowing effect occurred. After all the photolithography processes had been completed, the first OLED operation was confirmed by changing the applied voltages, resulting in a well illuminated blue light (Fig. 2d).

Fine patterning of the electrode was necessary if high-resolution display was to be achieved. An advantage of using photolithography process, as opposed to the shadow mask process, is the ability to accomplish fine patterning using the former. To verify this, we patterned the second and third anodes into 10 μm linewidths, using photolithography, and optical microscopy images for the patterned electrodes, and images from each OLED unit created by applying voltages, are shown in Fig. 2e, f, respectively. The linewidth of the electrode and the distance between the electrodes were both 10 μm. Figure 2e shows an image after second anode patterning was completed on the first OLED. In the yellow square box of Fig. 2e, the second anode is well aligned with the first anode. Figure 2f shows luminous images of *B*, *G*, and *R* OLED unit on the electrode patterned at 10 μm. These results established that photolithography process enabled fine patterning, and provided more uniform patterned area thickness than the shadow mask method.

### Performance of vertically stacked full-color OLEDs

To fabricate full-color OLEDs with vertically stacked *R*, *G*, and *B*, we conducted an optical simulation using a commercial software (SETFOS, Fluxim). In this simulation, we assumed that the orientation of the dipole was isotropic, and that it was located in the middle of each emitting layer (EML). The device structure for the optical simulation include: glass substrate (0.7 mm)/ITO (150 nm) as an anode of *B* unit/hexaazatriphenylenehexacarbonitrile (HAT-CN) (10 nm) as an hole injection layer (HIL) of *B* unit/hole transport layer (HTL)1 (*a* nm) of *B* unit/B host: B fluorescent dopant (5%, 10 nm) as an EML of *B* unit/electron transport layer (ETL)1 (*b* nm) of *B* unit/lithium fluoride (LiF) (1 nm) as an electron injection layer (EIL) of *B* unit/Al (2.5 nm)/Ag (30 nm) as a cathode of *B* unit/tris(8-hydroxyquinolinato)aluminum (Alq_3_) (60 nm) as a capping layer (CL)/Al_2_O_3_ (50 nm) as a TFE layer/SiN_x_ (150 nm) as a passivation layer for *B* unit/IZO (150 nm) as an anode of *G* unit/HAT-CN (10 nm) as an HIL of *G* unit/HTL2 (*c* nm) of *G* unit/PGH02 (refs.^44,45^) as a host for *R* and *G* dopants: G phosphorescent dopant (8%, 10 nm) as a EML of *G* unit/ETL2 (*d* nm) of *G* unit/LiF (1 nm) as an EIL of *G* unit/Al (2.5 nm)/Ag (30 nm) as a cathode of *G* unit/Alq_3_ (60 nm) as a CL/Al_2_O_3_ (50 nm) as a TFE layer/SiN_x_ (150 nm) as a passivation layer for *G* unit/IZO (150 nm) as an anode of *R* unit/HAT-CN (10 nm) as an HIL of *R* unit/HTL3 (*e* nm) of *R* unit/PGH02: R phosphorescent dopant (5%, 10 nm) as a EML of R unit/ETL3 (*f* nm) of *R* unit/LiF (1 nm) as an EIL of *R* unit/Al (100 nm) as a cathode.

**Fig. 3:**
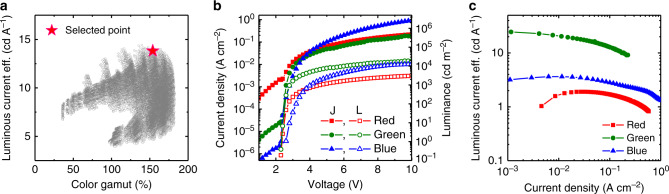
Optical simulation and characteristics of vertically stacked full-color organic light-emitting diodes (OLEDs). **a** Optical simulation results with 46,656 points plotted. **b** Current density (*J)–*voltage (*V*)*–*Luminance (*L*) characteristics, **c** Luminous current efficiency as a function of current density for independently controlled red, green, and blue OLEDs, in the vertically stacked full-color OLED.

The thicknesses of HTLs and ETLs of *R*, *G*, and *B* units can affect efficiency and color purity, due to electron–hole balance and micro-cavity effects. In this optical simulation, we changed the *R*, *G*, and *B* unit HTL and ETL thicknesses from 20 nm to 70 nm, through 10 nm step increment in this optical simulation. Figure 3a shows the simulation results with 46,656 points. Color gamut (sRGB) values were calculated using the color coordinates obtained from the optical simulation. The efficiency and color gamut values varied, depending on the *R*, *G*, and *B* unit HTL and ETL thicknesses. The conditions needed for high efficiency and for color gamut differed, as shown in Supplementary Table 2. The condition of maximum current efficiency and color gamut can reduce the electron–hole balance, due to significant differences between the HTL and ETL thicknesses. As an example, the HTL1 and ETL1 thickness difference was 50 nm for maximum color gamut. Considering current efficiency, color gamut, and electron–hole balance, we developed vertically stacked full-color OLEDs, using the fabricated device conditions listed in Supplementary Table 2, and shown as the red star in Fig. 3a. The fabricated vertically stacked OLED has a total thickness of 1.448 µm.

Figure 3b shows the *J–V–L* characteristics of the independently controlled R, G, and B units in the fabricated vertically stacked full-color OLEDs. The *R*, *G*, and *B* units exhibited typical OLED *J–V–L* characteristics, and the low turn-on voltages of *R*, *G*, and *B* unit were 2.3, 2.3, and 2.6 V, respectively. Each unit also had sufficient luminance for display applications; for instance, the *R*, *G*, and *B* units showed 710, 4,810, and 930 cd m^−2^, respectively, at 5 V. The *R* and *G* units had a slightly lower current than the *B* unit, which resulted from the relatively high sheet resistance of the IZO electrode compared with that of the ITO electrode. Figure 3c shows luminous current efficiency as a function of current density, and it can be observed that *R*, *G*, and *B* unit luminous current efficiencies were 1.9, 22.9, and 3.5 cd A^−1^, respectively, at ~1000 cd m^−2^. The *R* unit showed lower efficiency compared to the other units, which may have been due to optical loss, as the *R* units were placed on the uppermost layers of the full-color OLEDs.

**Fig. 4:**
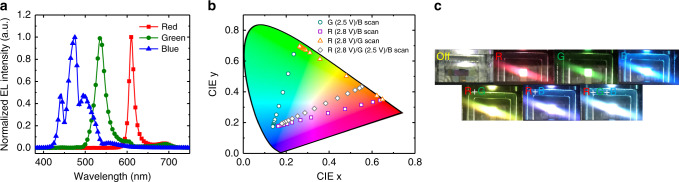
Color characteristics of vertically stacked full-color organic light-emitting diodes (OLEDs). **a** Normalized electroluminescence spectra for the independently controlled red (*R*), green (*G*), and blue (*B*) OLEDs. **b** Commission internationale de l’éclairage (CIE) 1931 color coordinates for three, independently controlled *R*, *G*, and *B* OLEDs, under fixed voltage and with other color unit voltage scanning. **c** Photographic images of vertically stacked full-color OLEDs with various colors.

Figure 4a shows the normalized EL spectra for independently controlled *R*, *G*, and *B* units, in the vertically stacked full-color OLEDs. The *B* unit EL spectrum exhibited a 475-nm main emission peak, with 497 nm and 442 nm shoulder peaks. The *G* and *R* unit EL spectra had 536 nm and 610 nm main peaks, respectively, with shoulder peaks that were very low—603 nm (*G*), 694 nm (*G*), and 691 nm (*R*)—owing to the micro-cavity effect. Each EL spectrum differed from its corresponding photoluminescence spectrum due to the micro-cavity effect. The *R* and *G* unit full-width-half-maximums (FWHMs) were markedly reduced, from 49 nm (*R*) and 71 nm (*G*) (see Supplementary Fig. 7), to 14 nm (*R*) and 25 nm (*G*), respectively, which are comparable to the FWHMs of quantum-dot EL devices. This narrow FWHM contributed to improving the color gamut of the vertically stacked full-color devices. The Commission internationale de l’éclairage (CIE) 1931 color coordinates for *R*, *G*, and *B* EL spectra were (0.664, 0.335), (0.271, 0.695), and (0.137, 0.176), respectively, and the calculated color gamut (sRGB) was approximately 112.7%.

Supplementary Fig. 8 shows the experiments to prove that there was little interference between the three colors, in this vertically stacked full-color OLED structure. The top-emitting *B* OLED was fabricated and the PGH02 50 nm/*R* emitter 10 nm was deposited on top of the cathode, to confirm the excitation of the *R* emitter by blue light, and the PGH02 50 nm/*G* emitter 10 nm were deposited to confirm the excitation of the *G* emitter by blue light. *B* OLED/PGH02 60 nm was fabricated as a control device. The detailed device structure is shown in Supplementary Fig. 8a, in which the *R* and *G* emitters represent PGH02:R dopant and PGH02:G dopant, respectively. Supplementary Fig. 8b shows that the *R* and *G* emitter absorption spectra overlap the control device emission spectrum; hence, excitation of the *R* and *G* emitters by blue light may occur. In Supplementary Fig. 8c, showing the logarithmic scale EL spectra, insignificant intensity was observed at 600–700 nm, for the *B* OLED/*R* emitter, and a slight tail at 550–600 nm was observed for the *B* OLED/*G* emitter. At first glance, *R* emitter or *G* emitter emission from reabsorption of blue light seemed to occur; however, considering that the *y*-axis is a logarithmic scale, the relative intensity was the order of 10^−3^, which was negligible. Therefore, based on the linear scale spectra in Supplementary Fig. 8d, there was almost no emission of *R* and *G* emitters due to excitation by blue light. The CIE color coordinates for the control device was (0.145, 0.038), which was almost unchanged with both the *R* emitter (0.144, 0.041), and the *G* emitter (0.144, 0.040; Supplementary Fig. 8e). In addition, the *B* OLED in the vertically stacked full-color OLED was designed for bottom emission, so that the amount of light going upwards, or directed to the *R* and *G* OLEDs, will be significantly less than the amount of light going downwards. Optical simulation was conducted to calculate the ratio of blue light depending on emission direction, with the results showing that the bottom emission was 10× more than the top emission. Therefore, both the optical simulation and experimental results implied that the excitation of *R* and *G* emitters by blue light was negligible in the vertically stacked full-color OLED.

Output color could be changed by controlling the *R*, *G*, and *B* unit driving voltages, as shown in Fig. 4b. For example, the green circle data points show color changes from green to blue, achieved by increasing the driving voltage of the *B* unit, in the presence of a fixed driving voltage in the *G* unit. The gray diamond data points were obtained when there was fixed driving voltages for the *R* (2.8 V) and *G* (2.5 V) units, respectively, and voltage scanning by the *B* unit. The color changed from yellowish-red to white, and then to blue. This showed that the device could produce any color inside the color space in response to changes to the *R*, *G*, and *B* unit driving voltages. As can be observed in Fig. 4c, the successful operation of the vertically stacked full-color OLEDs was demonstrated through the emission of various colors, such as red, green, blue, yellow (*R* + *G*), magenta (*R* + *B*), and white (*R* + *G* + *B*).

### TFT-driven vertically stacked full-color OLEDs

**Fig. 5:**
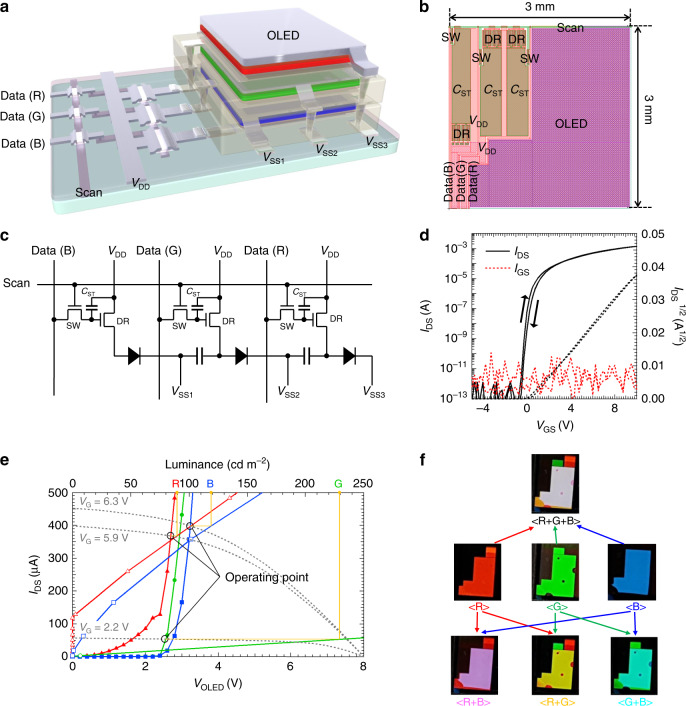
Thin-film transistor (TFT)-driven, vertically stacked full-color organic light-emitting diode (OLED). **a**, **b**, and **c** For the TFT-driven, full-color OLED with vertically stacked red (*R*), green (*G*), and blue (*B*), structure schematic, unit pixel layout, and equivalent circuit, respectively. **d** driving TFT transfer characteristics (black solid line) at a *V*_*DS*_ of 10 V. The red and black dashed lines indicate the gate-to-source leakage current (*I*_*GS*_) and square-root of drain-to-source current (*I*_DS_^*1/2*^), respectively. **e** Current characteristics for TFT-driven full-color OLED with vertically stacked R, G, and B pixels, where *V*_DD_ = 8 V, Data (*R*) = 5.9 V, Data (*G*) = 2.2 V, Data (*B*) = 6.3 V, and *V*_SS1_ = *V*_SS2_ = *V*_SS3_ = 0 V. The gray dotted lines are the output curves for the driving TFT, at *V*_G_ = 2.2 V, 5.9 V, and 6.3 V, corresponding to the data voltages. The solid colored lines with closed triangles, circles, and squares show the *I–V* characteristics for the *R*, *G*, and *B* units, respectively. The solid colored lines with open triangles, circles, and squares show current–luminance characteristics for the *R*, *G*, and *B* units, respectively. **f** Photographic images of the TFT-driven vertically stacked full-color OLEDs.

In Fig. 5a, the structure of the TFT-driven full-color OLED with vertically stacked *R*, *G*, and *B* units, is shown as a schematic. Due to the high processing temperature of the TFTs, they were all fabricated before being deposited on the OLED, as it was vulnerable to temperatures >100 °C. To drive the vertically stacked OLED, the driving (DR) TFTs should be connected to each OLED anode. Hence, the second and third anodes for the *G* and *R* units should be connected to the DR TFTs through via holes; detailed information on the via hole process is presented in Supplementary Fig. 9 and Supplementary Note 1). The unit pixel layout and equivalent circuits are shown in Fig. 5b, c, respectively. The two transistor-one capacitor (2T-1C) pixel structure was adopted to drive each OLED, while to increase the current driving capability, the DR TFT channel width/length (*W/L*) ratio was designed to be 980/10. The storage capacitor (*C*_*ST*_) needed to be sufficiently high, in relation to the parasitic capacitor formed by the overlap between the gate and source electrode of the switching (SW) TFT, to minimize the kickback voltage. The *C*_*ST*_ was designed to be 65 pF, which was ~200 times higher than the parasitic capacitor. The *W/L* ratio of the SW TFT was designed to be 100/10, to ensure sufficient charging time for our test platform active-matrix OLED, with a 60 Hz frame rate^46^. The aperture ratio for each OLED was 63%, which is a significant advantage of the vertically stacked OLED over a laterally arranged, three sub-pixel structure, where each aperture ratio cannot exceed 33.4%. This inherently high aperture ratio is beneficial in fabricating a high-brightness, long-lifetime, and high-resolution display.

Figure 5d shows the transfer curve of DR TFT at a drain-to-source voltage (*V*_DS_) of 10 V. The DR TFT exhibited low-leakage current, small hysteresis, and adequate on-current characteristics. The saturation mobility, subthreshold swing, and threshold voltage of the TFT were calculated to be 16.3 cm^2^ V s^−1^, 135 mV decade^−1^, and 0.29 V, respectively. Using these TFT characteristics, the vertically stacked R, G, and B units were operated as shown in Fig. 5e. The OLED currents (*I*_OLED_*s*) were determined by the gate voltages of the DR TFTs, which corresponded to the *R*, *G*, and *B* data voltages. Since the *V*_DS_ was equal to the supply voltage (*V*_DD_) minus the voltage applied to the OLED (*V*_OLED_), the *I*_OLED_ was determined by intersection of the current–voltage (*I–V*) curves of the OLEDs and output curves for the DR TFTs. For example, as shown in Fig. 5e, applying data voltages of 5.9, 2.2, and 6.3 V, respectively, for *R* (Data (*R*)), *G* (Data (*G*)), and *B* units (Data (*B*)), yielded approximately 90, 230, and 120 cd m^−2^ luminance respectively, for the *R*, *G*, and *B* units. By combining colors, the pixels could express red, green, blue, magenta (*R* + *B*), yellow (*R* + *G*), cyan (*G* + *B*), and white (*R* + *G* + *B*), as shown in Fig. 5f. In addition to these colors, the vertically stacked OLEDs could show full-color in a single-pixel, without sub-pixelation, as the *R*, *G*, and *B* unit currents could be controlled individually, which is attributed to the electrical isolation provided by the intermediate insulators. Note that conventional, vertically stacked full-color OLEDs share their intermediate electrodes with the bottom electrode of the upper-layer OLED and the top electrode of the lower-layer OLED^21,24–28^. As a result, when the voltage is adjusted to modulate a brightness of one color unit, the voltage of the adjacent OLEDs is affected as well, leading to a complicated driving method. In this study, the anodes and cathodes of each OLED were electrically separated, and each OLED was directly connected to each driving circuit, meaning that a common driving method of laterally arranged sub-pixel structures can be employed, even though the structure is vertically stacked.

## Discussion

A TFT-driven vertically stacked full-color OLED in a single-pixel, with a photolithography processed intermediate electrode was developed. Each OLED unit was vertically stacked and controlled independently. Unlike the conventional pixel structure of full-color displays, where the *R*, *G*, and *B* units are arranged laterally, this full-color OLED expresses all colors in a single pixel. This vertically stacked OLED can improve the low-geometric fill-factor of the conventional pixel system, which is a limitation for high-resolution display panels. In addition, by patterning the transparent intermediate electrode using photolithography processes, finer patterns can be achieved than are possible when using a shadow mask, which gets closer to high-resolution displays. Applying photolithography processes on top of the OLEDs was challenging, as OLEDs are vulnerable to moisture, oxygen, and organic solvents. We developed low-temperature Al_2_O_3_/SiN_x_ bilayered protection layer which stably protected the OLEDs, making photolithography processes on OLEDs possible. The onset voltage for all three *R*, *G*, and *B* units were found to be <3 V, and the individual OLED current efficiencies were 1.9, 22.9, and 3.5 cd A^−1^ for the *R*, *G*, and *B* units, respectively, at 1,000 cd m^−2^ luminance. The vertically stacked full-color OLED covered 112.7% of the sRGB color space, and the output color could be changed by controlling the *R*, *G*, and *B* unit driving voltages.

For the TFT-driven vertically stacked full-color OLED, an oxide TFT was employed, and the DR TFT was connected to each OLED anode through the via hole, to drive the full-color OLED. A 2T-1C pixel structure was adopted to drive each OLED unit. Due to geometrical advantage, the aperture ratio of the full-color-driven OLED pixel was determined to be approximately twice as large as conventional sub-pixel structures, in spite of TFT integration. By virtue of the photolithography-patterned intermediate insulators, a common driving method of laterally arranged sub-pixel structures could be employed, even though it was vertically stacked. We successfully demonstrated the TFT-driven full-color OLED with vertically stacked *R*, *G*, and *B* units, showing that the OLED was able to express a full range of colors in a single pixel. The device was stably operated after a few months when it was stored at ambient condition. In the respects of operational stability, since the OLED structure was designed to obtain high current efficiency and color purity, further research is required to improve operational lifetime and that is on the way.

This study has demonstrated the superior potential for high-resolution displays of over 2,000 pixels per inch by using OLEDs with high efficiency, fast response time, low weight, and high-color gamut. In fact, the technology developed in this work is more complicated than conventional color patterning processes, as vacuum deposition and solution processes are sequentially mixed, thereby making overall process costs high. Cost issues can be overcome by further development of solution processing technology, so that vacuum deposition can be replaced by solution processing for elements such as solution-processable electrodes, passivation layers, and light emitting devices. Since solution processing materials, devices, and fabrication processes have been recently develped and reported, we are optimistic that the future outlook for development of high-resolution displays using vertically stacked full-color OLEDs remains excellent.

## Methods

### Device fabrication

Glass substrates with patterned ITO were used for device fabrication. The substrates were sequentially cleaned with acetone, methanol, and deionized water, and then dried in a vacuum oven, at 80 °C. The organic, inorganic, and metal layers for the OLEDs were deposited using thermal evaporation. For TFE, 50 nm of Al_2_O_3_ was deposited by the atomic layer deposition (ALD) method at 95 °C. Subsequently, 150 nm of the SiN_x_ layer was deposited onto the Al_2_O_3_ layer using the PECVD method, at 100 °C. The 150-nm IZO layer was deposited as the intermediate electrode by RF sputtering, at 40 °C, and the IZO was patterned by the photolithography process. The fabricated OLEDs were transferred to an inert environment glove box, and they were encapsulated using a UV-curable epoxy, with a glass cap containing a moisture absorbent.

For the TFT-driven vertically stacked full-color OLED, oxide TFTs were fabricated using a reported method^47^. To fabricate the TFTs on glass substrates, 150-nm-thick Mo was first sputtered as a gate electrode. After the Mo patterning, 200 nm of SiO_2_ was deposited as a gate insulator, using PECVD. Subsequently, 30 nm of aluminum-doped In–Zn–Sn–O was sputtered and patterned as an active material, after which, 150 nm of Mo was sputtered and patterned as the TFT sources/drains. In all, 200 nm of SiO_2_ and 50 nm of Al_2_O_3_ were then sequentially deposited, by PECVD and ALD, respectively, for channel passivation. Subsequently, 150 nm of ITO was sputtered and patterned as an anode for the first OLED. To form a bank structure (fabricated to 2.5 μm height), an organic insulator (acrylic photoresist) was spin-coated and patterned, after which the TFTs were post-annealed, at 200 °C for 2 h, in a vacuum oven. All the materials were patterned by photolithography processes.

### Photolithography processes

Photolithography processes were conducted for both the via hole formation and IZO intermediate electrode patterning. Chrome-coated glass was used for photomasks. Positive PR was spin-coated onto the samples, and soft baking was carried out, at 90 °C for 90 s. A Karl Suss MA6 Contact mask aligner was used for aligning the photomask with samples. After UV exposure, PR development and hard baking (at 100 °C for 3 min) were carried out. For via hole formation, samples were transferred to a helical etcher (nexso NSE-8100). SiN_x_ was etched using CF_4_/O_2_-mixed gas (RF source, 200 W; RF bias, 70 W), and Al_2_O_3_ was etched using Cl_2_/Ar-mixed gas (RF source, 300 W; RF bias, 100 W). For patterning the IZO intermediate electrode, the wet etch process was performed, at room temperature, using IZO etchant solution (a mixture of nitric acid and hydrochloric acid). After the etching process was completed, residual PR was removed, at room temperature, by dipping the device in PR strip solution (N-methyl-2-pyrrolidinone containing 2-ethoxyethyoxy ethanol solution). Inspection was carried out using an optical microscope.

### Measurement and characterization

Transmittance was measured on a UV/VIS/NIR spectrometer (PerkinElmer Lambda 750). The refractive index at 633 nm was measured using a spectroscopic ellipsometer (M2000D by Woollam), and SEM images were measured on a Sirion 400. AFM images were measured using a scanning probe microscope (XE-100; Park Systems). To confirm the water vapor transmission rate of the alumina layer, we used a commercial MOCON Aquatran device (Model 2; MOCON Inc.), operating at 37.8 °C and 100% relative humidity. Current density–voltage characteristics were measured using a Keithley-238 source–measure unit. Luminance and EL spectra were obtained using a spectroradiometer (CS-2000; Konica Minolta). All measurements were taken in a dark room, at room temperature. To operate the R-, G-, and B-units independently, three source–measure units (Keithley-238, Keithley-2400, and Keithley-6517A) were used.

## Supplementary information

Supplementary Information

Supplementary Movie 1

Description of Additional Supplementary File

## Data Availability

The authors declare that all data supporting the findings of this study are available within the paper and its supplementary information files or from the corresponding author upon reasonable request.
