# Supplementary material for: Thin-film transistor-driven vertically stacked full-color organic light-emitting diodes for high-resolution active-matrix displays
Source: Nat Commun. 2020 Jun 1;11:2732. doi: 10.1038/s41467-020-16551-8 (PMC7264127; doi:10.1038/s41467-020-16551-8)
Supplement: Supplementary file 1 — Supplementary Information [file 41467_2020_16551_MOESM1_ESM.pdf]

Supplementary Information

Thin-film transistor-driven vertically stacked full-color organic light-emitting diodes for high-resolution active-matrix displays

Choi *et al*

Supplementary Figures

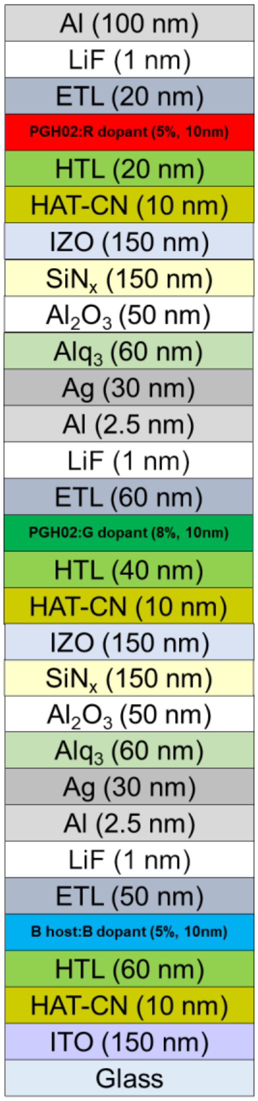

Supplementary Figure 1 | Schematic structure of vertically stacked full-color OLED. Detailed schematic structure and layer information of the vertically stacked full-color OLED.

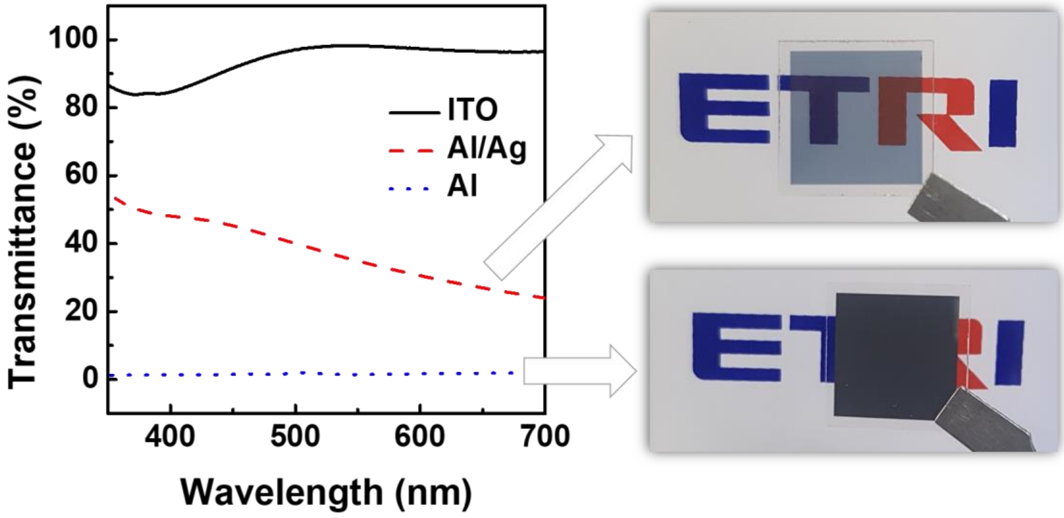

Supplementary Figure 2 | Transmittance of electrodes. Transmittance spectra for the ITO, Al / Ag, and Al, with the photographic images of the semi-transparent intermediate cathodes (Al 2.5 nm / Ag 30 nm) for the B and G units, and the reflecting cathode (Al 100 nm) for the R unit.

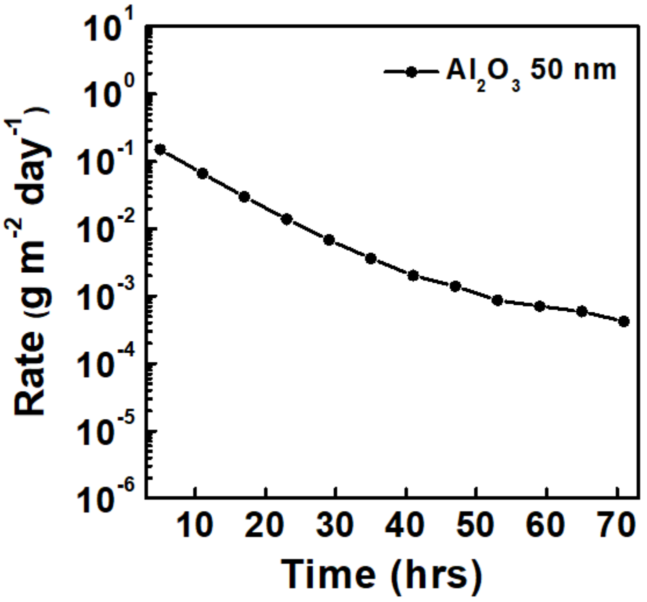

Supplementary Figure 3 | Water vapor transmission rate (WVTR) property. The WVTR value of the 50-nm-thick Al_2O_3 layer at 37.8 ^{o}C and 100 % relative humidity.

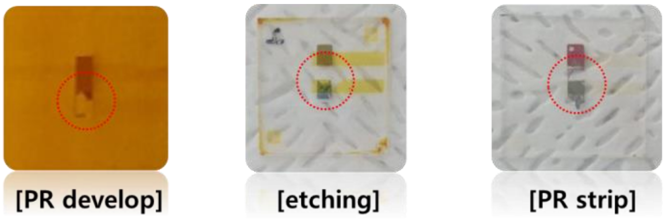

Supplementary Figure 4 | Chemical resistance of OLED. The photograph images of the damaged devices (OLED/TFE) after each photolithography processes.

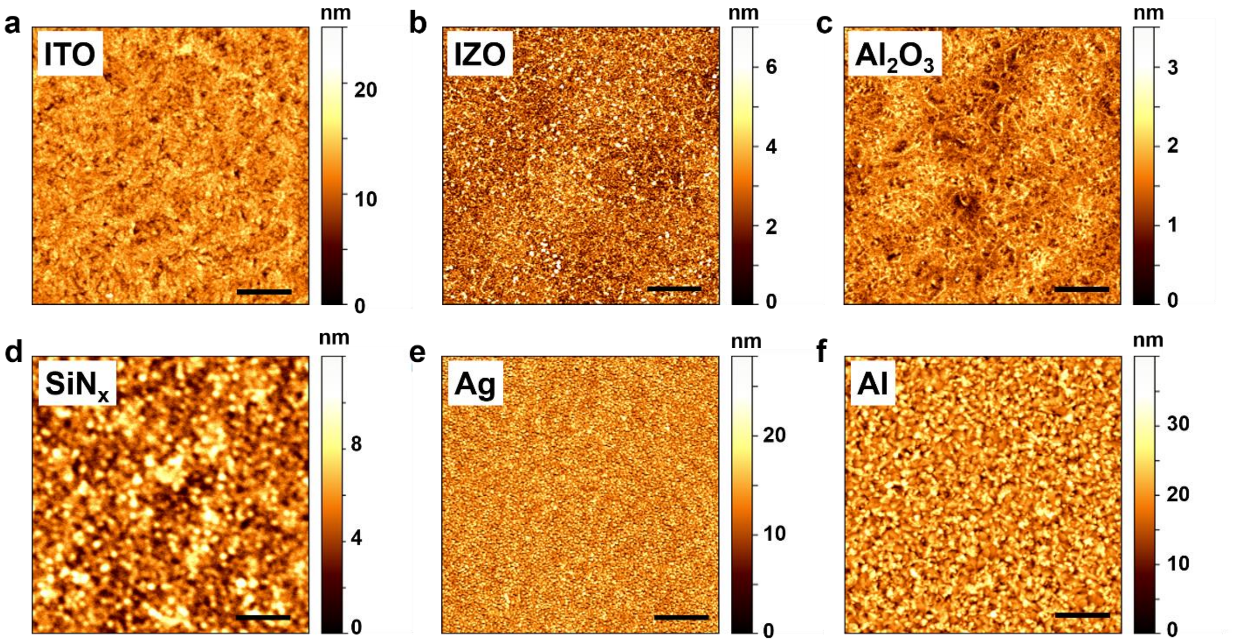

Supplementary Figure 5 | Surface morphologies of intermediate layers and electrodes. The AFM images of the ITO, IZO, Al_2O_3, SiN_x, Ag, and Al layers. Scale bar, 1 μm.

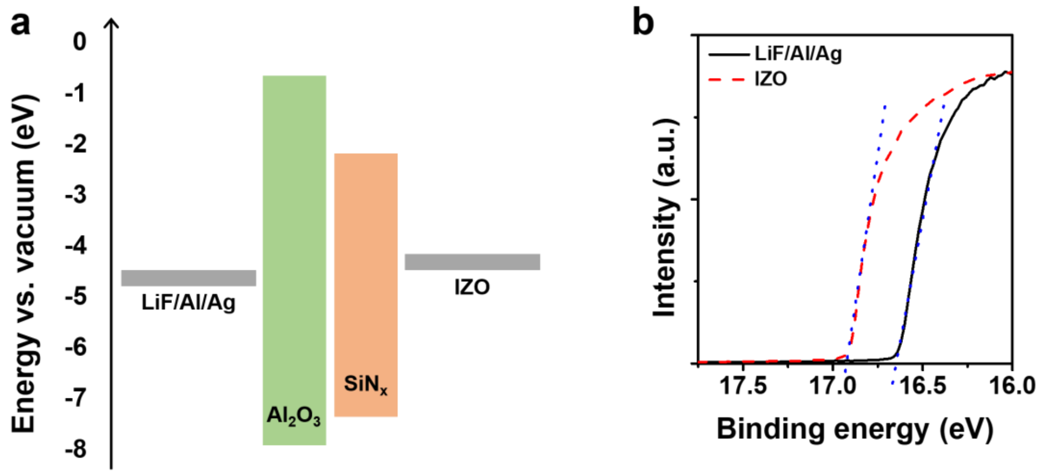

Supplementary Figure 6 | Energy level of intermediate layers. The energy level diagram of intermediate layers (Al_2O_3 and SiN_x) and adjacent electrodes (LiF / Al / Ag and IZO). **b** UPS spectra for LiF / Al / Ag and IZO, in the cut off region.

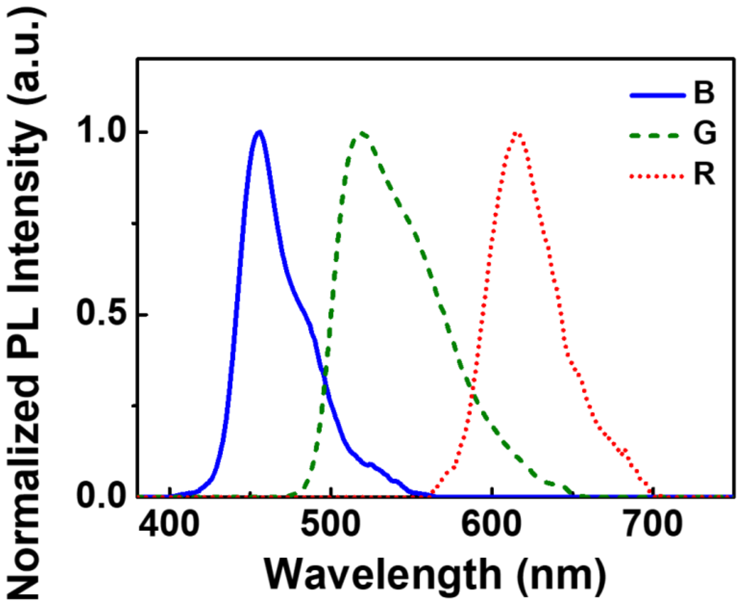

Supplementary Figure 7 | Photoluminescence spectra of B, G, and R dopants.

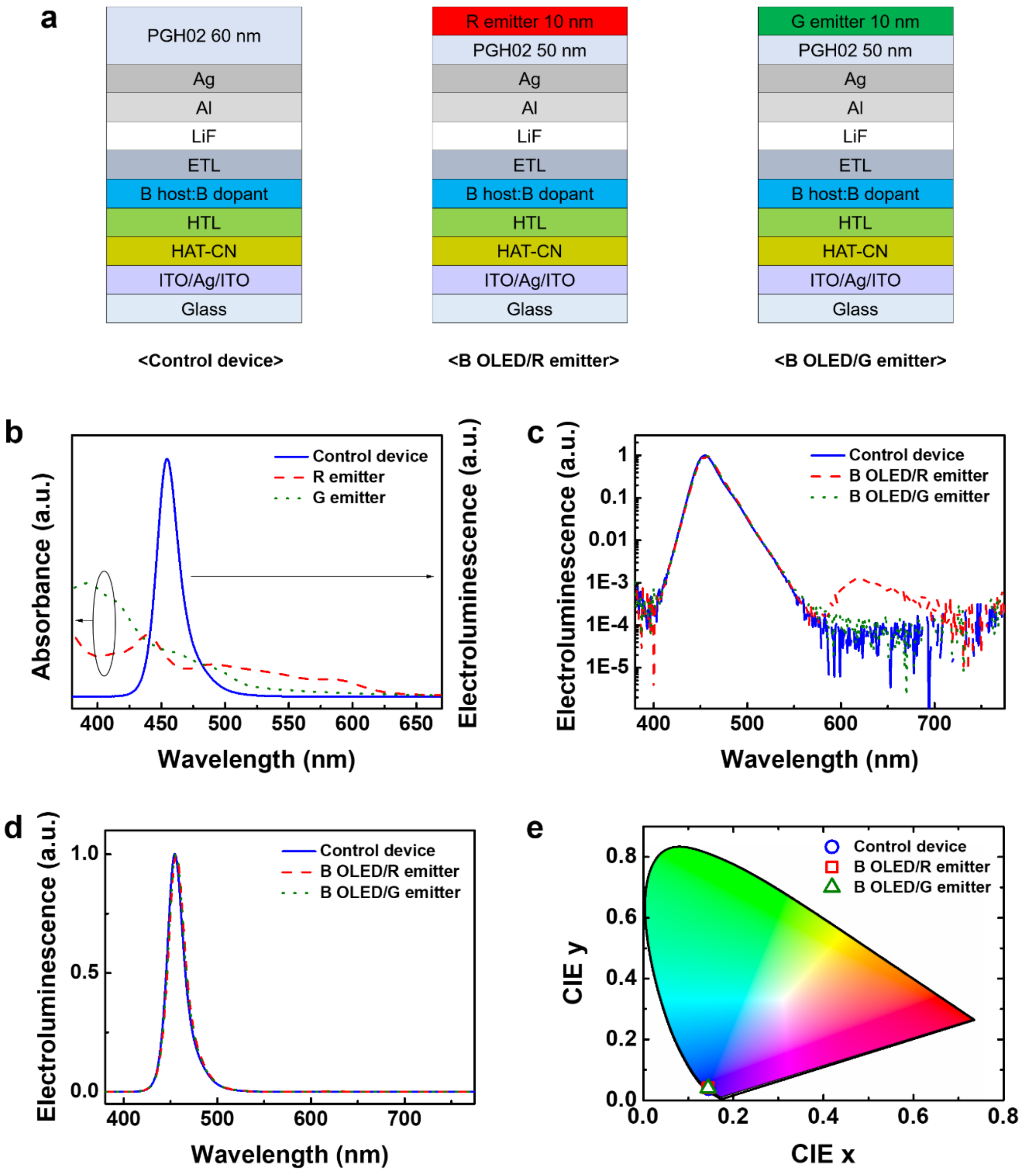

Supplementary Figure 8 | Experiments to prove that there was little interference between the three colors in the vertically stacked full-color OLED. a The device schematics. **b** The absorption spectra for the R emitter (red dashed line), and G emitter (green dotted line), and electroluminescence (EL) spectrum of the control device (blue solid line). **c** The logarithmic scale EL spectra for the control device (solid blue line), B OLED/R emitter (red dashed line), and B OLED/G emitter (green dotted line). **d** The linear scale EL spectra for the control device (solid blue line), B OLED/R emitter (red dashed line), and B OLED/G emitter (green dotted line). **e** The CIE coordinates for the three devices—the control device, B OLED/R emitter, and B OLED/G emitter.

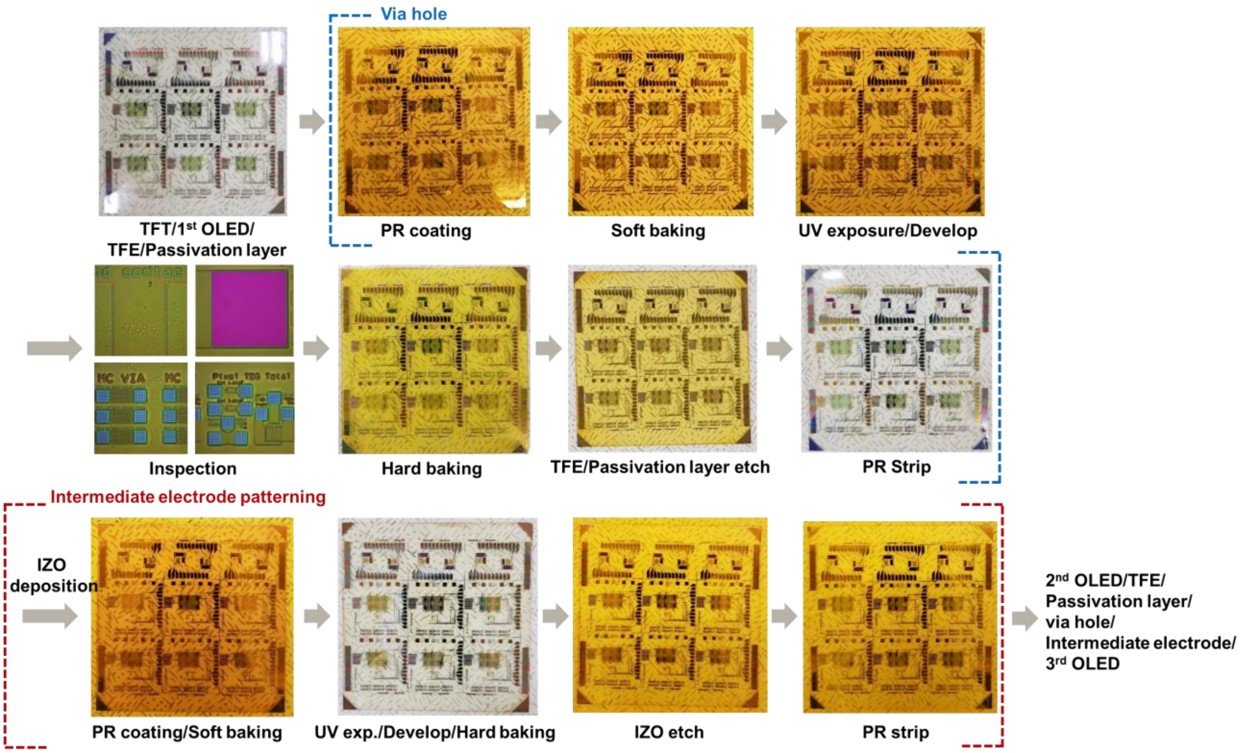

Supplementary Figure 9 | Photographic images of TFT-driven vertically stacked full-color OLED for each process step.

Supplementary Tables

	ITO	IZO	Al_2O_3	SiN_x	Ag	Al
**R_q (nm)**	2.0	1.2	0.4	1.7	3.0	4.6

Supplementary Table 1 | Root-mean-square roughness (R_q) value of electrodes (ITO, IZO, Ag, and Al) and intermediate layers (Al_2O_3, and SiN_x).

Condition	HTL1 (nm)	ETL1 (nm)	HTL2 (nm)	ETL2 (nm)	HTL3 (nm)	ETL3 (nm)	LCE (R+G+B) (cd A^{-1})	Color gamut (sRGB) (%)
**Maximum LCE**	70	50	30	60	70	70	14.4	139.6
**Maximum color gamut**	70	20	20	50	20	30	9.5	182.7
**Fabricated device**	60	50	40	60	20	20	13.8	153.8

Supplementary Table 2 | Optical simulation results. The HTL and ETL thicknesses of each R, G, and B unit for maximum luminous current efficiency (LCE), maximum color gamut, and the fabricated device.

Supplementary Note

Supplementary Note 1 | Processing steps for TFT-driven vertically stacked full-color OLED.

Supplementary Figure 9 shows the photographic images of TFT-driven vertically stacked full-color OLED for each process step. Via hole is needed to connect the TFT with the electrodes of OLEDs. Seven steps in blue dashed bracket is photolithography processes for via hole formation. Desired part of TFE and passivation layer were etched by mixed gases (see methods for detailed information). After that, for intermediate electrodes of second OLED unit, IZO was deposited and patterned by photolithography processes (4 images in red dashed bracket in Supplementary Figure 9). Then, G OLED as a second OLED unit was deposited on the patterned IZO electrodes, and TFE/passivation layer was successively deposited. After that, the processes of via hole formation and intermediate patterning were repeated for third OLED unit.
