# Supplementary material for: Thin-film transistor-driven vertically stacked full-color organic light-emitting diodes for high-resolution active-matrix displays
Source: Nat Commun. 2020 Jun 1;11:2732. doi: 10.1038/s41467-020-16551-8 (PMC7264127; doi:10.1038/s41467-020-16551-8)
Supplement: Supplementary file 3 — Description of Additional Supplementary File [file 41467_2020_16551_MOESM3_ESM.docx]

**Description of Additional Supplementary File**

**File Name**: Supplementary Movie 1

**Description**: A video for successful operation of thin-film transistor-driven vertically stacked full-color OLEDs. By combining colors, the pixel can express red, green, blue, yellow (red+green), magenta (red+blue), cyan (green+blue), and white (red+green+blue).
